# Allometric Scaling of the Active Hematopoietic Stem Cell Pool across Mammals

**DOI:** 10.1371/journal.pone.0000002

**Published:** 2006-12-20

**Authors:** David Dingli, Jorge M. Pacheco

**Affiliations:** 1 Division of Hematology, Mayo Clinic College of Medicine Rochester, Minnesota, United States of America; 2 Program for Evolutionary Dynamics, Harvard University Cambridge, Massachusetts, United States of America; 3 Centro de Física Teórica e Computacional e Departamento de Física da Faculdade de Ciências Lisboa, Portugal; Sanofi-Aventis, United States of America

## Abstract

**Background:**

Many biological processes are characterized by allometric relations of the type *Y* = *Y*_0_ *M^b^* between an observable *Y* and body mass *M*, which pervade at multiple levels of organization. In what regards the hematopoietic stem cell pool, there is experimental evidence that the size of the hematopoietic stem cell pool is conserved in mammals. However, demands for blood cell formation vary across mammals and thus the size of the active stem cell compartment could vary across species.

**Methodology/Principle Findings:**

Here we investigate the allometric scaling of the hematopoietic system in a large group of mammalian species using reticulocyte counts as a marker of the active stem cell pool. Our model predicts that the total number of active stem cells, in an adult mammal, scales with body mass with the exponent ¾.

**Conclusion/Significance:**

The scaling predicted here provides an intuitive justification of the Hayflick hypothesis and supports the current view of a small active stem cell pool supported by a large, quiescent reserve. The present scaling shows excellent agreement with the available (indirect) data for smaller mammals. The small size of the active stem cell pool enhances the role of stochastic effects in the overall dynamics of the hematopoietic system.

## Introduction

Scaling, in physics, has provided fundamental insights from the tiny quark to the universe at large. In biology, allometric scaling [1] is typically associated with a simple power law: *Y* = *Y*_0_ *M^b^*, where *Y* is some observable and *M* is the mass of the organism. Allometric scaling has been identified on a wide range of observables [2]–[5], from basal metabolic rate (**BMR**), heart rate, aortic and tree trunk radii, to unicellular genome lengths. Intriguingly, most of the scaling exponents *b* are multiples of the power ^1^/_4_, and the origin of this general dependence has been subjected to intense investigation. A beautiful explanation was proposed by West et al. [4], where the ^3^/_4_ exponent in **BMR** scaling was related to a surface to volume ratio in a four-dimensional biological world. Although other explanations have been proposed [6], the ^3^/_4_ scaling has been challenged [7], [8] and plants have recently been found to scale differently [9], allometric scaling remains ubiquitous in biology, and has provided novel insights on various biological processes.

**Figure 1:**
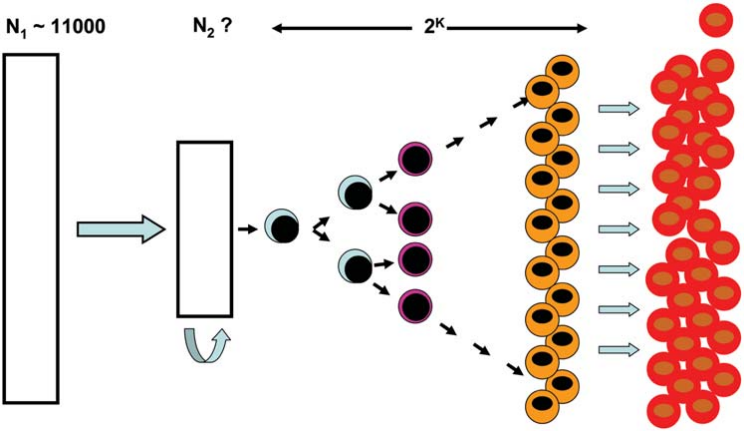
Schematic representation of the current understanding of erythropoiesis. Hematopoietic stem cells exist in two distinct compartments: cells in N_2_ are actively replicating and contributing to blood cell formation. They are supported by a larger quiescent pool (N_1_). Cell replication leads to an exponential amplification (2^K^) of progeny that ultimately differentiate into the various types of cells present in the circulation.

Blood is also ubiquitous, and has been the subject of investigation for centuries. Our present understanding of the hematopoietic system has improved tremendously since William Harvey described the circulatory system in the 17^th^ century. Indeed, our current understanding relies on the concept of stem cells [10]–[15]. This view identifies the bone marrow as the site of active cell replication, responsible for the maintenance of the circulating blood cell pool that is continuously undergoing apoptotic senescence. Blood cell production is maintained by hematopoietic stem cells that have the capacity to both self-renew and differentiate into all types of blood cells [12] (see Figure 1). The number of hematopoietic stem cells has been estimated in various species using indirect methods. These include the SCID repopulating cell (SRC) assay [16], rescue of lethally irradiated mice and rats using serial dilutions of bone marrow derived cells [17], [18] and ferrokinetic studies that estimate red blood cell production and correlate this with total marrow cellularity and the myeloid to erythroid ratio [19].

Perhaps rather surprisingly, Abkowitz et al. [19] recently suggested that the total number of hematopoietic stem cells is conserved from mouse to humans, and proposed that this may apply to all mammals. They estimated that mammals have between 11000 and 22000 hematopoietic stem cells. Subsequent studies in *Rattus norvegicus* were consistent with this prediction [18]. There is indirect evidence to suggest that this may also be true in elephants, whose African variant may reach a mass of approximately 6000 kg [20]. However, the demands for blood cell production vary across species: the number of cells produced by a murine bone marrow over the life-time of a mouse is equal to what a human bone marrow produces in a day or a cat in a week [21].

Potential mechanisms that can explain these different production rates include (i) an active stem cell pool that varies with animal size, (ii) faster rates of replication as the size of the species increases, (iii) longer cell specific contribution to hematopoiesis and (iv) a number of committed cell lineages that produce terminally differentiated blood cells which increases with animal size (Figure 1), with none of these mechanisms being mutually exclusive.

Current models of hematopoiesis suggest that stem cells exist in two functionally distinct compartments [22]: a quiescent, reserve pool where cells divide rarely, and another pool of actively replicating cells that contribute to hematopoiesis for some time to be replaced by cells from the reserve pool. Since the demands for blood cell production vary across species [17]–[19], [21], it is possible that the active stem cell pool scales allometrically with adult mass despite the fact that the total number of stem cells may be conserved across mammalian species [19].

Potential explanations (i) and (ii) relate the hematopoietic system with animal size. Basal metabolism is clearly related to the hematopoietic system since hemoglobin, exclusively transported by red blood cells, is the main carrier of oxygen throughout the organism, thereby ensuring an adequate supply for metabolic needs. Consequently, it is natural to assume the existence of allometric scaling in the hematopoietic system, a feature which may prove insightful in determining the size and properties of the stem-cell pool.

In the following, we make use of allometric scaling to estimate the number of active stem cells contributing to hematopoiesis in adult mammals from voles to elephants.

### Allometric scaling of the active stem-cell pool

As is well known, blood volume is a function of animal mass [23]. Furthermore, rates of cell production in an animal should scale, under normal conditions, with the species specific **BMR** [3]. The specific **BMR** -*R_M_*- has been shown to scale as *R_M_*∼*M* ^−1/4^ across 27 orders of magnitude, from the sub-cellular respiratory complexes to unicellular organisms up to the largest mammals [3]. If red blood cells of a given animal originate from *N_SC_* active stem cells, then we may expect that, per unit time, the number of red blood cells produced will scale as *N_SC_R_M_*. During hematopoiesis, stem cells divide and as the daughter cells become more differentiated, they progressively lose their ability to replicate. However, during replication the number of cells produced from one stem cell increases exponentially at an ideal rate of 2*^k^*, where *k* represents the number of distinct replication steps before the cell loses the ability to divide (Figure 1). Here we assume that the process exhibits the same efficiency across species and as such contributes with a constant factor to red blood cell production.

Red blood cell *production* is best evaluated by measuring the total number of circulating reticulocytes (*R_T_*) in a given species. Indeed, estimating blood *production* based on red blood cell *survival* may be misleading, since many extrinsic factors contribute to premature red blood cell destruction, and these can be difficult to quantify [24]. On the other hand, circulating reticulocytes, precursors of red blood cells, should reflect more accurately blood *production* originating from the active stem cell pool. Consequently, we predict that reticulocyte production per day (*R_TD_*) scales as *R_TD_*∼*N_SC_R_M_*.

*R_TD_* can be obtained by dividing *R_T_* by the time required for reticulocyte maturation, *τ*. In other words, *R_TD_*∼*R_T_τ* ^−1^∼*R_T_R_M_* since, under normal conditions, the specific **BMR** *R_M_* ultimately determines maturation time [25]. Equating the two expressions for *R_TD_* allows us to predict that *N_SC_*∼*R_T_*. Therefore, we can deduce the allometric scaling of the active stem cell pool in the animal's bone marrow from the corresponding scaling of the total reticulocyte count across species. In Materials and Methods we describe how we collected data for *R_T_* from mammalian species, as well as the procedure used to determine its allometric exponent.

## Results and Discussion

By fitting the data collected for reticulocyte count across different mammalian species in the way described in the Materials and Methods section, we obtained a ¾ power law- scaling for the mass-dependence of the size of the active stem-cell pool, *N_SC_ ∼ M* ^3/4^ Using the well-studied cat data [26] as a reference, which estimates the size of the active stem cell pool as *N_SC_* = 13 after a stem cell transplant, the allometric relation above predicts that *N_SC_* = 111 in humans (mass  =  70 kg). This result is very similar to the reported value *N_SC_* = 116 of active stem cells after bone marrow transplantation in humans [27]. Under normal physiologic conditions, the active stem cell pool in the cat is composed of *N_SC_*≥40 cells [26]. Using the minimum value, the allometric relation leads to an active pool size of *N_SC_*≈385 cells for humans. This result is very similar to estimates based on observation on adult women heterozygous for chronic granulomatous disease (CGD) [28]. Furthermore, the present allometric scaling relation also predicts that one single stem cell is enough to maintain hematopoiesis for the entire lifespan of a mouse, consistent with published estimates [21]. The overall consistency between the predictions based on the allometric relation and available (though scarce) data for mice, cats and humans suggests that allometric scaling provides a powerful tool to investigate the overall properties of the hematopoietic stem cell pool. On the other hand, such an overall consistency also provides additional support to the underlying assumptions made in the derivation of the allometric scaling relation. Extrapolating our data for the pilot whale (*Globicephala macrorhynchus*, mass ∼2250 kg) we estimate *N_SC_* = 4690 whereas for the Asian elephant (*Elephas maximus*, mass ∼6000 kg) *N_SC_* = 9640 cells, both of which are below the minimum number (11000) of total stem cells that are thought to be present in any mammal [19].

According to the Hayflick hypothesis, stem cells replicate a finite number of times before senescence [29]. Associating the replication rate of stem cells with *R_M_* means the Hayflick hypothesis is equivalent to state that the lifespan of a stem cell scales with *R*_M_^−1^∼M^1/4^. In the laboratory experiments of Ref. [26] it was found that stem cells replicate, on average, once every 2.5 weeks in mice and once every 8.3 to 10 weeks in cats (after transplant). These estimates follow the *M* ^3/4^scaling relation we derived. Extrapolating these observations to humans, we obtain a replication rate of once every ≈20 weeks, significantly lower than the measurements in Ref. [30] (1 year) or the extrapolation made in Ref. [26] (42 weeks). Note however that this latter number was estimated assuming from the outset that stem cells replicate 100 times during the lifetime of a human (80 years). In contrast, the existence of allometric scaling is the only requirement of our theory, from which the relevant number can be deduced without further assumptions. Furthermore, *N_SC_* = 111 results from allometric scaling for post-transplant conditions, which do not reflect normal adult hematopoiesis. If we take the allometric prediction for normal conditions - *N_SC_* = 385 - then we arrive at a replication value of approximately once every 60 weeks, close to the results based on an analysis of telomere shortening in human hematopoietic cells [30]. The present estimates are consistent with the idea that mammals do not exhaust their hematopoietic stem cell reserve during their lifetime. Indeed, if we assume that a cell replicates 100 times before senescence, then the lifetime of a murine stem cell is longer than the lifetime of a mouse, consistent with the view [21] that a single stem cell may be enough to maintain hematopoiesis for the entire murine lifespan. In fact, our present allometric relation predicts that, although larger mammals require larger active stem cell pools, the expected lifetime of each species-specific stem cell is always longer than the expected lifetime of the mammal.

In retrospect, examining the possible explanations invoked in the beginning of this work, clearly argument (i) was given a precise allometric justification, whereas (ii) can be ruled out, since it would contradict the well-established allometric scaling of the specific **BMR** [3] which suggests that the rate of replication of cells decreases as the mass of the mammal increases. Argument (iii) derives from the Hayflick hypothesis and is supported by our estimates. The available data does not allow us to elaborate on argument (iv), but we can state that no special adaptations seem necessary to explain hematopoiesis in whales or elephants.

A ¾ scaling of *N_SC_* has been rationalized in terms of a surface-to-volume ratio in the four biological dimensions [4], [5]. This calls for detailed studies of the structure-function relationships in the bone marrow. Indeed, the above scaling is consistent with the interpretation of the bone marrow as a heterogeneous microenvironment in which the active stem cell pool is distributed in surface niches [31], whereas the quiescent stem cell pool might presumably occupy the bulk of the bone marrow, in accord with the prevailing view of the stem cell pool as metaphorically compartmentalized [22]. As such, detailed studies of bone marrow physiology may shed light on the mechanisms responsible for the ¾ scaling relations which pervade in living systems. Finally, the reduced size of the active stem cell pool in mice, cats and humans indicates that stochastic effects may play a sizeable role in the overall dynamics of the hematopoietic system in these mammals. Such effects will be explored elsewhere [32].

## Materials and Methods

**Figure 2:**
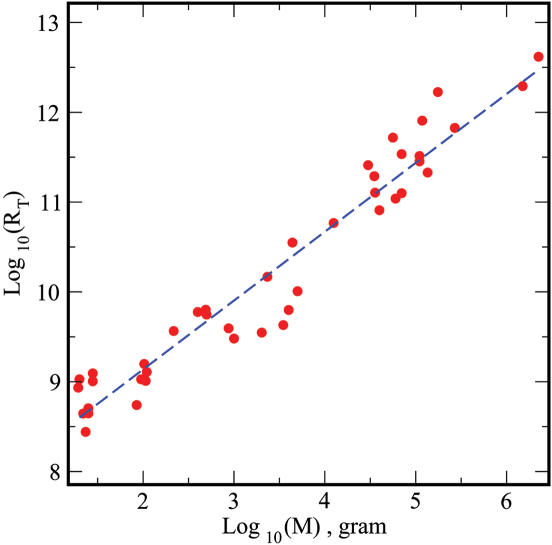
Allometric scaling of reticulocyte count. We plot the logarithm of reticulocyte count of a given mammal as a function of the logarithm of its mass (in gram). Plotted data correspond to a total of 40 mammal species with masses ranging over 6 orders of magnitude, from voles to whales. The data corresponds to the mean value of the reticulocyte count for each species. A straight linear fit leads to a coefficient of 0.745, remarkably close to the ¾ exponent (R^2^ = 0.94, p<0.0001, CI 0.70–0.76), with an intercept of 7.641. Hence for the smallest mammal (a shrew with ∼3 gram), we predict a reticulocyte count of ∼1×10^8^.

The total number of circulating reticulocytes was calculated as the product of the concentration of circulating reticulocytes in the blood and the blood volume of mature animals for each species. In Figure 2 we plot the logarithm of *R_T_* as a function of the logarithm of the mass, for mammals that cover over six orders of magnitude. The range varied from voles with a mass of 20 g up to a pilot whale with an average mass of 2250 kg. The data corresponds to the mean value of the reticulocyte count for each species (detailed minimum and maximum counts are provided in Table S1). The dashed blue line was obtained from a linear regression, which leads to an exponent of 0.745≈¾ (*R* ^2^ = 0.94, *p*<0.0001). The confidence intervals limit the allometric exponent between 0.70 and 0.76. The intercept of the fitting curve at a mass of 1 gram is 7.641. Hence, we estimate a value of ∼10^8^ for the reticulocyte count of the smallest mammal (a shrew with a mass of 3 g).

## Supporting Information

Table S1 Upper and lower limit of the logarithm of circulating reticulocytes across mammals.
(0.07 MB DOC)
