# Supplementary material for: Allometric Scaling of the Active Hematopoietic Stem Cell Pool across Mammals
Source: PLoS One. 2006 Dec 20;1(1):e2. doi: 10.1371/journal.pone.0000002 (PMC1762381; doi:10.1371/journal.pone.0000002)
Supplement: Table S1 — Upper and lower limit of the logarithm of circulating reticulocytes across mammals. (0.07 MB DOC) [file pone.0000002.s001.doc]

**Table S1. Upper and lower limit of the logarithm of circulating reticulocytes across mammals**

**Order Family Species Mass (g) Log Ret lo Log Ret Hi Reference**

Rodentia Muridae *Microtus agrestis* 20 8.99 9.06 [1]

Rodentia Muridae *Microtus agrestis* 20 8.97 9.08 [1]

Rodentia Muridae *Mus musculus*  22 8.09 8.88 [2]

Rodentia Muridae *Mus musculus*  25 8.11 8.88 [2]

Rodentia Muridae *Mus musculus*  25 8.15 8.94 [2]

Rodentia Muridae *Mesocricetus auratus* 85 8.0 9.0 [3]

Rodentia Muridae *Mesocricetus auratus* 103 9.5 [4]

Rodentia Muridae *Mesocricetus auratus* 107 8.26 9.27 [3]

Rodentia Muridae *Mesocricetus auratus* 110 9.41 [4]

Rodentia Muridae *Rattus norvegicus*  490 9.49 9.98 [5]

Rodentia Muridae *Rattus norvegicus*  400 9.34 9.99 [5]

Rodentia Muridae *Rattus norvegicus*  500 9.08 10.0 [5]

Rodentia Caviidae *Cavia porcellus* 1000 9.78 [6]

Rodentia Sciuridae *Spermophilus parryii* 217.7 9.49 9.63 [7]

Dasyuromorpha Dasyuridae *Antechinus stuartii* 23.3 8.385 8.491 [8]

Dasyuromorpha Dasyuridae *Sminthopsis crassicaudata* 28 8.51 9.23 [9]

Dasyuromorpha Dasyuridae *Sminthopsis crassicaudata* 28 8.928 9.21 [9]

Dasyuromorpha Dasyuridae *Sminthopsis macroura* 19.35 8.84 9.01 [9]

Dasyorumorpha Marmosidae *Marmosa mitis* 95 8.97 9.08 [10]

Diprodontia Phalangeridae *Trichosurus vulpecula* 2020 9.359 9.676 [11]

Lagomorpha Leporidae *Oryctolagus cuniculus* 2330 9.977 10.3 [12]

Lagomorpha Leporidae *Oryctolagus cinuculus* 4400 10.38 10.67 [12]

Carnivora Felidae *Felis catus*  3500 8.98 9.88 [13]

Carnivora Felidae *Felis catus* 4000 9.14 10.05 [14]

Carnivora Canidae *Canis familiaris* 12500 9.68 11.05 [15]

Carnivora Canidae *Canis familiaris*  12670 8.82 8.9 [16]

Carnivora Canidae *Canis familiaris*  30000 10.15 11.7 [17]

Carnivora Canidae *Canis familiaris*  35000 10.2 11.57 [18]

Carnivora Phocidae *Phoca vitulina* 110000 11.31 11.56 [19]

Carnivora Otariidae *Zalophus californianus* 270000 11.53 12.0 [19]

Cetacea Delphinidae *Cephaloehychus commersonii* 56000 11.4 11.9 [19]

Cetacea Delphinidae *Delphinus delphis* 118000 11.75 12.02 [19]

Cetacea Odontoceti *Tursiops truncates* 175000 11.31 12.5 [19]

Cetacea Monodontidae *Delphinapterus leucas* 1500000 11.77 12.52 [19]

Cetacea Delphinidae *Globicephala macrorhynchus* 2250000 12.45 12.74 [19]

Artiodactyla Suidae *Sus scrofa* 35656 10.18 11.38 [20]

Artiodactyla Suidae *Sus scrofa* 110000 10.58 11.79 [21]

Primates Cercopithecinae *Macaca mulatta* 5000 9.73 10.176 [22]

Primates Cebidae *Saimiri sciureus* 875 8.48 9.88 [23]

Primates Cercopithecidae *Papio sp*  40000 11.21 [24]

Primates Pongidae *Pan troglodytes* 60000 11.34 [25]

Primates Hominidae *Homo sapiens sapiens* 70000 11.13 11.74 [26]

Primates Pongidae *Pongo pygmaeus* 70000 11.40 [27]

Primates Pongidae *Gorilla gorilla* 135000 11.63 [28]

Ret lo = Lower limit of reticulocyte count

Ret hi = Upper limit of reticulocyte count

**References**

1. Newson J, Chitty, D. (1962) Haemoglobin levels, growth and survival in two Microtus populations. Ecology 43: 733-738.

2. Moore DM (2000) Hematology of the mouse (Mus musculus). Schalm's Veterinary Hematology Fifth Edition: 1219-1224.

3. Moore DM (2000) Hematology of syrian (golden) hamster (Mesocricetus auratus). Schalm's Veterinary Hematology Fifth Edition.

4. Lyman CP, Weiss LP, O'Brien RC, Barbeau AA (1957) The effect of hibernation on the replacement of blood in the golden hamster. J Exp Zool 136: 471-485.

5. Moore DM (2000) Hematology of the rat (Rattus norvegicus). Schalm's Veterinary Hematology Fifth Edition: 1210-1218.

6. Moore DM (2000) Hematology of the guinea pig (Cavia porcellus).. Schalm's Veterinary Hematology Fifth Edition: 1107-1110.

7. Barker JM, Boonstra R (2005) Preparing for winter: divergence in the summer-autumn hematological profiles from representative species of the squirrel family. Comp Biochem Physiol A Mol Integr Physiol 142: 32-42.

8. Cheal PD, Lee, A.K., Barnett, J.L. (1976) Changes in the haematology of Antechunus stuartii (Marsupialia) and their association with male mortality. Australian Journal of Zoology 24: 299-311.

9. Haynes JI, Skidmore, G.W. (1991) Haematology of the dasyurid marsupials Sminthopsis crassicaudata and Sminthopsis macroura. Australian Journal of Zoology 39: 157-169.

10. Wolf HG, Sifrine, M., Klein, A.K., Foin, A.T. (1971) Hematologic values for laboratory reared Marmosa mitis. Laboratory Animal Science 21: 249-251.

11. Presidente PJA, Correa, J. (1981) Haematology, plasma electrolytes and serum biochemical values of Trichosurus vulpecula (Kerr) (Marsupialia: Phalangeridae). Australian Journal of Zoology 29: 507-517.

12. Moore DM (2000) Hematology of rabbits. Schalm's Veterinary Hematology Fifth Edition: 1100-1106.

13. Clinkenbeard KD, Meinkoth, J. (2000) Normal hematology of the cat. Schalm's Veterinary Hematology Philadephia: Lipincott Williams & Wilkins: 1064-1068.

14. Jain NC (1986) The cat: normal hematology with comments on response to disease. Schalm's Veterinary Hematology: Philadelphia: Lea & Febiger.

15. Michaelson SM, Scheer K, Gilt S (1966) The blood of the normal beagle. J Am Vet Med Assoc 148: 532-534.

16. Beckman DA, Evans JW, Oyama J (1978) Studies on the erythron and the ferrokinetic responses in beagles adapted to hypergravity. Aviat Space Environ Med 49: 1331-1336.

17. Lumsden JH, Mullen K, McSherry BJ (1979) Canine hematology and biochemistry reference values. Can J Comp Med 43: 125-131.

18. Meinkoth JH, Clinkenbeard, K.D. (2000) Normal hematology of the dog. Schalm's Veterinary Hematology Fifth Edition: 1057-1063.

19. Reidarson TH, Duffield, D., McBain, J. (2000) Normal hematology of marine mammals. Schalm's Veterinary Hematology Fifth Edition: 1164-1173.

20. Burke JD (1954) Blood volume in mammals. Physiological Zoology 26: 1-21.

21. Thorn CE (2000) Normal hematology of the pig. Schalm's Veterinary Hematology Fifth Edition: 1089-1095.

22. Stahl WR, Malinow MR (1967) A survey of physiological measurements in Macaca mulatta. Folia Primatol (Basel) 7: 12-33.

23. Ausman LM, Gallina DL, Hayes KC, Hegsted DM (1976) Hematological development of the infant squirrel monkey (Saimiri sciureus). Folia Primatol (Basel) 26: 292-300.

24. Hack CA, Gleiser, C.A. (1982) Hematologic and serum reference values for adult and juvenile baboons (Papio sp.). Laboratory Animal Science 32: 502-505.

25. Burns KF, Ferguson, F.G., Hampton, S.H. (1967) Compedium of normal blood values for baboons, chimpanzees, and marmosets. Am J Clin Pathol 48: 484.

26. Beutler E (2001) Production and destruction of erythrocytes. Williams Hematology Sixth Edition: 355-368.

27. McClure HM (1972) Hematologic and blood chemistry data for the orangutan (Pongo pygmaeus). Folia Primatol (Basel) 18: 284-299.

28. McClure HM (1972) Hematologic and blood chemistry data for the gorilla (Gorilla gorilla). Folia Primatol (Basel) 18: 300-316.
